# The association between outdoor air pollution and body mass index, central obesity, and visceral adiposity index among middle-aged and elderly adults: a nationwide study in China

**DOI:** 10.3389/fendo.2023.1221325

**Published:** 2023-10-09

**Authors:** Wei Pan, Menglong Wang, Yingying Hu, Zhengqi Lian, Haonan Cheng, Juan-Juan Qin, Jun Wan

**Affiliations:** ^1^Department of Cardiology, Renmin Hospital of Wuhan University, Wuhan, China; ^2^Cardiovascular Research Institute, Wuhan University, Wuhan, China; ^3^Hubei Key Laboratory of Cardiology, Wuhan, China; ^4^Department of Geriatrics, Zhongnan Hospital of Wuhan University, Wuhan, China; ^5^Center for Healthy Aging, Wuhan University School of Nursing, Wuhan, China; ^6^Dong Fureng Institute of Economic and Social Development, Wuhan University, Wuhan, China

**Keywords:** obesity, body mass index, waist circumference, visceral adiposity index, air pollution, epidemiologic study

## Abstract

**Background:**

Previous animal studies have suggested that air pollution (AP) exposure may be a potential risk factor for obesity; however, there is limited epidemiological evidence available to describe the association of obesity with AP exposure.

**Methods:**

A retrospective cross-sectional study was conducted on 11,766 participants across mainland China in 2015. Obesity was assessed using body mass index (BMI), waist circumference (WC), and visceral adiposity index (VAI). The space-time extremely randomized tree (STET) model was used to estimate the concentration of air pollutants, including SO_2_, NO_2_, O_3_, PM_1_, PM_2.5_, and PM_10_, matched to participants’ residential addresses. Logistic regression models were employed to estimate the associations of obesity with outdoor AP exposure. Further stratified analysis was conducted to evaluate whether sociodemographics or lifestyles modified the effects.

**Results:**

Increased AP exposure was statistically associated with increased odds of obesity. The odds ratio (ORs) and 95% confidence interval (CI) of BMI-defined obesity were 1.21 (1.17, 1.26) for SO_2_, 1.33 (1.26, 1.40) for NO_2_, 1.15 (1.10, 1.21) for O_3_, 1.38 (1.29, 1.48) for PM_1_, 1.19 (1.15, 1.22) for PM_2.5_, and 1.11 (1.09, 1.13) for PM_10_ per 10 μg/m^3^ increase in concentration. Similar results were found for central obesity. Stratified analyses suggested that elderly participants experienced more adverse effects from all 6 air pollutants than middle-aged participants. Furthermore, notable multiplicative interactions were found between O_3_ exposure and females as well as second-hand smokers in BMI-defined obesity.

**Conclusions:**

This study suggested that outdoor AP exposure had a significant association with the risk of obesity in the middle-aged and elderly Chinese population. Elderly individuals and women may be more vulnerable to AP exposure.

## 1 Introduction

Obesity has been a persistent and alarming public health issue for the past few decades (1). It is considered a chronic, progressive, relapsing disease that substantially elevates the risk of various comorbidities, including fatty liver disease, type 2 diabetes mellitus, myocardial infarction, hypertension, and stroke, leading to a reduced quality of life and a shortened life expectancy (2). A recent study suggested that the age-standardized prevalence of obesity has been rising steadily from 1980 to 2015 in every country in the world (3); specifically, the obesity prevalence increased with age from 20 years and reached its peak at the age of 50 to 65 years. In addition, the prevalence of obesity was generally higher in women than in men, and the maximal sex difference was observed in middle-aged and elderly adults. In summary, obesity has now replaced smoking, becoming the leading lifestyle-related risk factor for premature mortality (2), and imposes a considerable economic burden on society as a whole.

The increasing prevalence of obesity can be attributed to lifestyle and environmental changes that have accompanied rapid advancement in industrialization and urbanization worldwide, especially in many Asian countries (4). Epidemiological studies have shown that air pollution (AP), mainly due to emissions from vehicular traffic and industrial processes (5), is related to the risk of obesity. Studies have demonstrated that maternal exposure to particulate matter with aerodynamic diameters of ≤ 2.5 μm (PM_2.5_) was linked to a higher birth weight of newborns (6, 7); in parallel, long-term exposure to ambient AP in children has been connected to a higher body mass index (BMI) and increased likelihood of obesity (8, 9). In adults, previous studies also provided evidence that exposure to AP was correlated with increased BMI and central obesity (10–12). In addition, experimental models in animals have provided possible biological plausibility that AP exposure could be a crucial risk factor for obesity and its complications (13–15). However, due to rapid industrialization and urbanization in recent years, China is facing the dual problems of population aging and environmental pollution. Moreover, since China has a vast territory and complex climate, resulting in large differences in AP levels in different regions, there is limited systematic epidemiological evidence describing the association between AP and obesity in middle-aged and elderly adults at a representative nationwide level, not only prohibiting the understanding of any potential mechanism for the association between AP and obesity but also hampering accurate estimates of the burden of obesity.

This study investigates the relationship between AP exposure and obesity in middle-aged and elderly individuals within the Chinese population. All participants were enrolled from the third follow-up of the China Health and Retirement Longitudinal Study (CHARLS).

## 2 Materials and methods

### 2.1 Study design and participants

This cross-sectional study obtained data from the CHARLS dataset, which is a national longitudinal cohort study that enrolled middle-aged and elderly adults in China, currently consisting of individuals aged 45 years and above, as well as their spouses or children when possible. The baseline survey of CHARLS consecutively employed a multistaged, stratified probability-proportionate-to-size sampling method to enroll respondents, which covered 450 villages in 28 provinces, 150 counties, and districts across mainland China. The baseline survey recorded sociodemographics, lifestyles, and health status through computer-assisted in-person interviews and subsequently assessed participants’ anthropometric measurements and biosamples, including blood and urine. Details about the recruitment method and designs of the CHARLS have been published previously (16).

The current study included the third follow-up of CHARLS participants in 2015, including a total of 13,337 respondents who had complete blood biochemistry data. Exclusion criteria included i) participants whose ages were under 45 (n = 1,150) and ii) participants with incomplete information on indoor fuel or missing anthropometric measurements (n = 421); the study finally included 11,766 individuals in the entire population dataset.

### 2.2 Assessment and definition of obesity

In the current study, BMI, waist circumference (WC), and visceral adiposity index (VAI) were all adopted to define obesity. VAI, an estimate of visceral adiposity that combines lipid parameters and anthropometric measurements, has been noted to be superior to general anthropometric parameters when predicting the occurrence of cardiovascular disease and metabolic syndrome (17, 18). Anthropometric measurements (height, weight) of each participant were recorded in accordance with WHO guidelines (19). Briefly, a stadiometer was used to measure height, and participants stood upright with their back against the vertical backboard of the stadiometer and kept their head in the Frankfort horizontal plane position. A scale was used to record weight, and participants stood with their shoes off (20). WC was measured with a soft measuring tape at the level of the navel. After obtaining anthropometric measurements, the participants’ BMI was computed using the standard formula of weight in kilograms divided by the square of height in meters. According to the guidelines for the prevention and control of obesity in the Chinese population (21), the obese state was defined by BMI cutoff value (obesity, BMI ≥ 28, non-obesity, BMI< 28). The modified WC cutoffs for Chinese populations were used to define central obesity (22), where WC ≥ 90 cm for men and ≥ 80 cm for women. VAI was calculated using a sex-specific formula according to a previous study and divided into quartiles when performing analysis (23), where triglyceride (TG) and high-density lipoprotein cholesterol (HDLc) levels were expressed in mmol/L:


, $$Male:\;VAI=\;\left(\frac{WC}{39.68+1.88\times BMI}\right)\times \left(\frac{TG}{1.03}\right)\;\times \left(\frac{1.31}{HDL}\right)$$

$$Female:VAI=\;\left(\frac{WC}{36.58+1.89\times BMI}\right)\times \left(\frac{TG}{0.81}\right)\;\times \left(\frac{1.52}{HDL}\right)$$

### 2.3 Measurement of individual AP exposure

We collected annual concentrations of sulfur dioxide (SO_2_), nitrogen dioxide (NO_2_), ozone (O_3_), and particulate matter with aerodynamic diameters of ≤ 1 μm (PM_1_), PM_2.5_, and PM_10_ levels at 10 km spatial resolution from the ChinaHighAirPollutants (CHAP) dataset. The method was described previously (24, 25). Briefly, the space-time extremely randomized tree (STET) model was utilized to estimate ambient AP concentrations in this study, which incorporates satellite remote sensing with machine learning techniques that account for the spatiotemporal variation in AP. The estimations were highly in accordance with surface measurements, as evidenced by average cross-validation coefficient of determination values (CV-R^2^) of 0.84, 0.93, 0.87, 0.77, 0.92, and 0.90 and root-mean-square error (RMSE) values of 10.07, 4.89, 17.10, 14.6, 10.76, and 21.12 μg/m^3^ for the daily concentrations of SO_2_, NO_2_, O_3_, PM_1_, PM_2.5_, and PM_10_, respectively.

Individual annual exposures to SO_2_, NO_2_, O_3_, PM_1_, PM_2.5_, and PM_10_ were geographically matched based on county-level longitude and latitude data. To minimize the disparities in air pollution exposure resulting from individuals’ outdoor work or commuting activities, exposures of AP assigned to each individual were determined as the average air pollution concentrations within a 15 km radius around their corresponding latitude and longitude coordinates. Then, 1-year average exposures for each participant in CHARLS wave 3 were employed in the current study.

### 2.4 Assessment and definition of covariates

Several potential confounders were controlled in the current study, including sociodemographics, lifestyles, health status and meteorological factors. Meteorological factors, such as temperature and specific humidity, were collected from the China Meteorological Administration’s meteorological monitoring stations (available at http://data.cma.cn/). Other confounding factors were collected *via* standardized questionnaires by experienced interviewers. In this study, sociodemographic covariates were sex (male and female), age level, which was categorized as middle-aged (45 to 64 years) and elderly (65 years and above), residence, which was grouped as rural and urban, and education attainment, grouped as “middle school or below” and “high school or above” (**Supplementary Materials**). Lifestyle-related covariates included smoking status, categorized as non-smoker, smoker, and second-hand smoker. In relation to smoking status, the term “smoker” referred to a person who had ever smoked before, while “second-hand smoker” was defined as someone who had never smoked himself but had family members who did; alcohol consumption, which was categorized into 3 groups: non-drinker, drink but less than once a month and drink more than once a month; indoor fuel use, which was classified as either “clean fuel use” or “solid fuel use”. Clean fuel use included solar energy, natural gas, biogas, liquefied petroleum gas, and electricity, while solid fuel use included coal, crop residue, and wood burning. Participants who never cooked and those who used other fuels were excluded according to previous study (26). Health status covariates included a self-reported history of hypertension and diabetes mellitus.

### 2.5 Statistical analysis

The baseline characteristics of the participants were described based on their obesity status. Continuous variables were examined by Kruskal−Wallis tests and presented as the median and the interquartile range (IQR). Categorical variables were presented as counts (percentages) and examined by Chi-squared tests. This study utilized a multilayered analysis strategy. Logistic regression models were performed to estimate the odds ratio (ORs) and 95% confidence interval (CI) of obesity in relation to outdoor AP exposure (per 10 μg/m^3^ increase in each air pollutant level). The crude estimates were present in Model 0. Based on Model 0, Model 1 was further adjusted for age at baseline visit and sex. Based on Model 1, Model 2 was additionally adjusted for sociodemographic factors (education attainment), lifestyle-related risk factors (alcohol consumption and smoking status), and indoor fuel use.

To investigate potential effect modification by sociodemographic or lifestyle factors, we conducted stratified analyses of the fully adjusted model (Model 2) by age, sex, education attainment, alcohol consumption, and smoking status. In each stratification, except for variables used for stratification, all other covariates were controlled in the models.

To evaluate the reliability of our results, we also conducted several sensitivity analyses *via* the following methods: a. including only participants who had resided at their present residential location for over 3 years, b. including meteorological factors (temperature and specific humidity) as covariates, c. including history of hypertension and diabetes mellitus as covariates, and iv) excluding participants with a personal history of cardiac events or stroke. A logistic regression-based restricted cubic spline model was implemented to assess the potential nonlinear associations between 6 air pollutants and obesity measured on a continuous scale. To strike a balance between model fit and overfitting of the primary spline for obesity, the knot value corresponding to the minimum Akaike information criterion (AIC) within the knot range from 3 to 5 was selected. Subsequently, variance analysis was conducted to test the statistical significance of the nonlinear relationships. The maximum ORs of obesity on the spline curves correspond to the air pollutant concentrations with the highest risk.

R version 4.2.1 was used for all statistical analyses in this study. Cases with missing covariate values were imputed by the nonparameter method *missForest* algorithm (27). Statistical significance in this study was defined as a p value less than 0.05 (two-tailed).

## 3 Results

### 3.1 Participant descriptive characteristics and AP exposure

**Figure 1:**
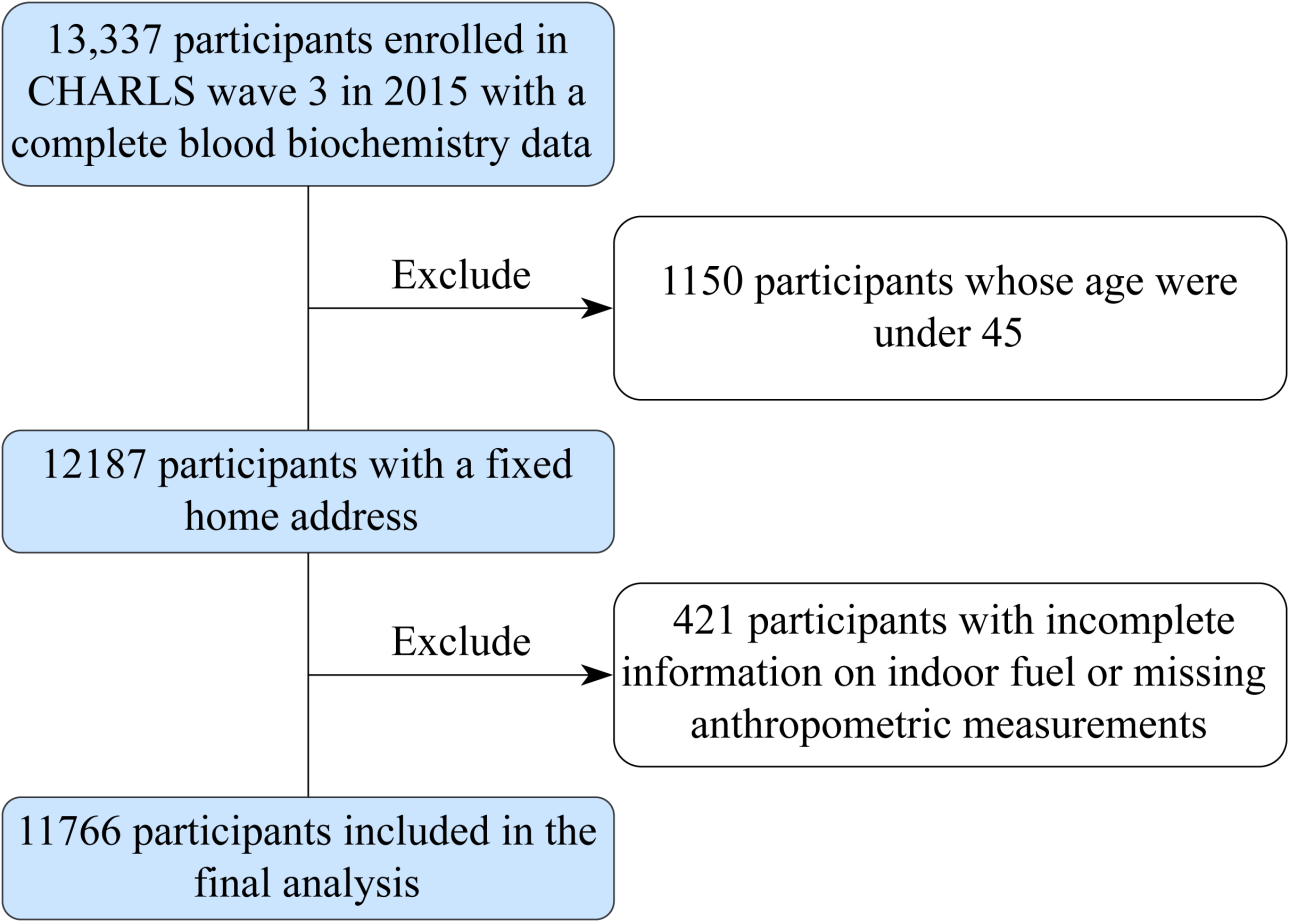
Schematic flow chart of patient inclusion in the study. CHARLS, the China Health and Retirement Longitudinal Study.

**Figure 2:**
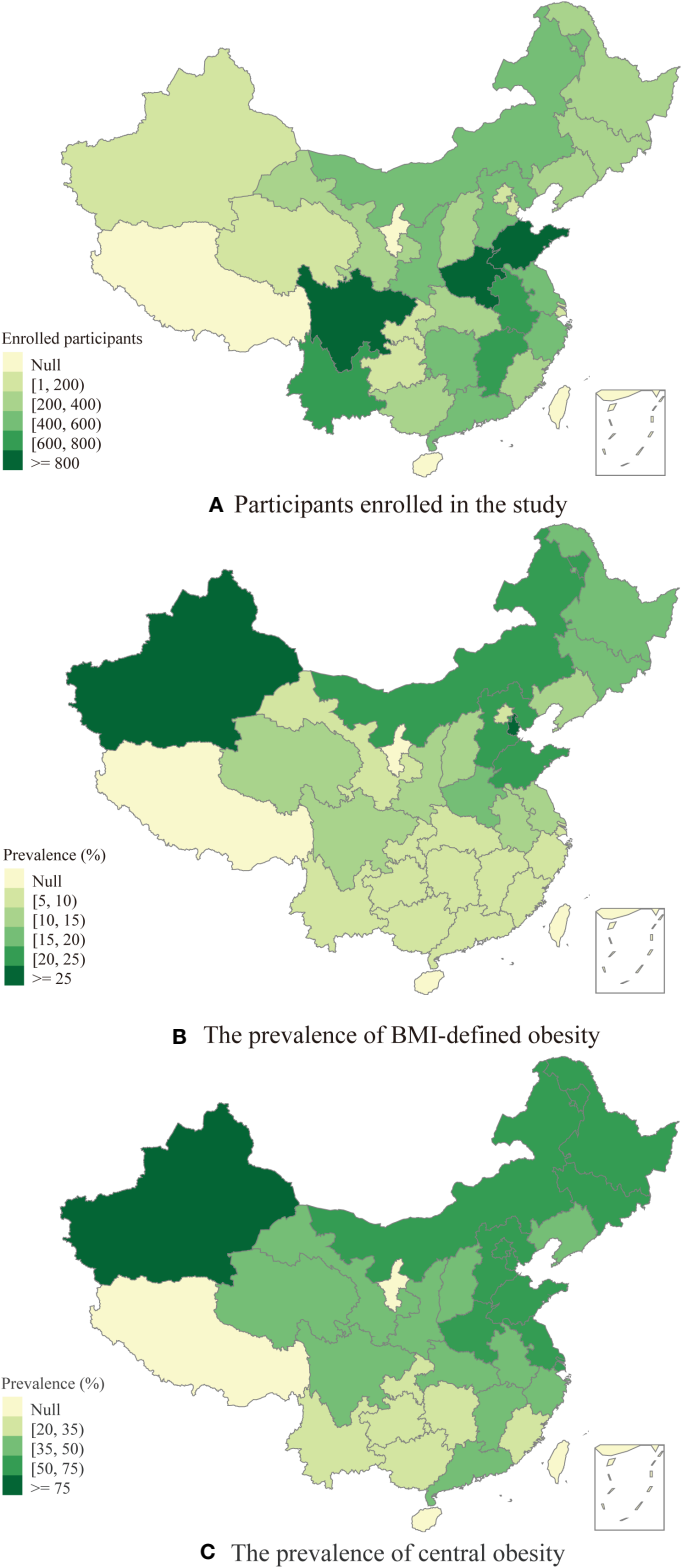
**(A)** The distribution of participants enrolled in the study. **(B)** The prevalence of BMI-defined obesity among middle-aged and elderly people. **(C)** The prevalence of central obesity among middle-aged and elderly people.

In the current study, we analyzed a total of 11,766 middle-aged and elderly individuals (see **Figure 1**). These respondents were enrolled from 445 communities located in 125 counties across 28 Chinese provinces. **Figure 2** displays the geographical distribution of the study participants and the prevalence of obesity. The overall BMI-defined obesity prevalence was 13.3% (1,561/11,766). The prevalence of BMI-defined obesity in all provinces ranges from 5.88% to 26.87%. Tianjin, Xinjiang and Shandong had the top three prevalence rates at 26.87%, 25.93% and 21.92%, respectively (**Supplementary Table 1**). Regarding spatial distribution patterns, the prevalence of BMI-defined obesity in eastern China was generally higher than that in other regions, particularly in the northeastern regions, and lower in the southern and central regions. Xinjiang, a western province, also maintained a higher prevalence than other regions.

**Table 1** shows the characteristics of individuals categorized by BMI-defined obesity. The respondents had a median age of 60 years old, 52.8% were female, and 61.94% lived in rural areas. Participants diagnosed with obesity tended to be slightly younger (57 vs. 60 years, P<0.001) and had a higher probability of being female (63.55%, P<0.001) and second-hand smokers (39.65%, P<0.001). In the current analysis, the median SO_2_, NO_2_, O_3_, PM_1_, PM_2.5_, and PM_10_ concentrations were 23.95 μg/m^3^ (range 16.37-36.22), 30.22 μg/m^3^ (range 21.84-38.12), 81.43 μg/m^3^ (range 74.94-92.30), 35.38 μg/m^3^ (range 31.53-41.08), 52.20 μg/m^3^ (range 39.62-64.34), and 86.69 μg/m^3^ (range 62.68-106.49), respectively. A similar distribution of the basic characteristics of participants grouped by WC-defined central obesity was observed and presented in **Supplementary Table 2**.

**Table 1:** Characteristics of the participants according to the presence of BMI-defined obesity.

Characteristics^a^	level	Overall (n = 11,766)	Non-obesity (n = 10,205)	Obesity (n = 1,561)	p value
Demographics					
Age, years		60.00 [52.00, 67.00]	60.00 [52.00, 67.00]	57.00 [51.00, 64.00]	<0.001*
Age group	Middle-aged (45 - 64 years)	7985 (67.87)	6781 (66.45)	1204 (77.13)	<0.001*
	Elderly (65 years and above)	3781 (32.13)	3424 (33.55)	357 (22.87)	
Gender	Male	5553 (47.20)	4984 (48.84)	569 (36.45)	<0.001*
	Female	6213 (52.80)	5221 (51.16)	992 (63.55)	
Education level	Middle school or below	8989 (88.01)	7797 (87.88)	1192 (88.82)	0.346
	High school or above	1225 (11.99)	1075 (12.12)	150 (11.18)	
Residence	Rural	7288 (61.94)	6418 (62.89)	870 (55.73)	<0.001*
	Urban	4478 (38.06)	3787 (37.11)	691 (44.27)	
Lifestyle behaviors					
Smoking Status	Non-smoker	3132 (26.62)	2675 (26.21)	457 (29.28)	<0.001*
	Smoker	4877 (41.45)	4392 (43.04)	485 (31.07)	
	Second-hand Smoker	3757 (31.93)	3138 (30.75)	619 (39.65)	
Drinking Status	Non-drinker	7617 (64.74)	6504 (63.73)	1113 (71.30)	<0.001*
	Drink but less than once a month	1033 (8.78)	911 (8.93)	122 (7.82)	
	Drink more than once a month	3116 (26.48)	2790 (27.34)	326 (20.88)	
Clinical characteristics					
SBP, mmHg		126.67 [114.33, 141.33]	125.33 [113.67, 140.33]	132.67 [121.67, 145.67]	<0.001*
DBP, mmHg		74.67 [67.67, 82.67]	74.00 [67.00, 81.67]	79.33 [72.00, 87.00]	<0.001*
Pulse, bpm		73.00 [66.33, 80.00]	73.00 [66.33, 80.00]	74.33 [67.58, 81.00]	<0.001*
BMI, kg/m^2^		23.67 [21.35, 26.25]	23.09 [20.94, 25.09]	29.64 [28.73, 31.25]	<0.001*
Waistline, cm		86.05 [79.00, 93.40]	84.40 [78.00, 90.60]	101.20 [96.50, 105.60]	<0.001*
Laboratory measures					
FBG, mmol/L		5.31 [4.90, 5.91]	5.31 [4.90, 5.81]	5.51 [5.11, 6.31]	<0.001*
Tch, mmol/L		4.68 [4.12, 5.32]	4.67 [4.10, 5.30]	4.83 [4.26, 5.50]	<0.001*
TG, mmol/L		1.30 [0.94, 1.93]	1.25 [0.91, 1.83]	1.76 [1.25, 2.64]	<0.001*
LDL, mmol/L		2.61 [2.14, 3.10]	2.59 [2.13, 3.08]	2.69 [2.23, 3.20]	<0.001*
HDL, mmol/L		1.29 [1.12, 1.49]	1.31 [1.13, 1.51]	1.20 [1.05, 1.35]	<0.001*
Comorbidities					
Hypertension	No	8508 (72.31)	7664 (75.10)	844 (54.07)	<0.001*
	Yes	3258 (27.69)	2541 (24.90)	717 (45.93)	
Diabetes	No	10895 (92.60)	9524 (93.33)	1371 (87.83)	<0.001*
	Yes	871 (7.40)	681 (6.67)	190 (12.17)	
Cardiac disease	No	10193 (86.63)	8972 (87.92)	1221 (78.22)	<0.001*
	Yes	1573 (13.37)	1233 (12.08)	340 (21.78)	
Stroke	No	11547 (98.14)	10028 (98.27)	1519 (97.31)	0.012*
	Yes	219 (1.86)	177 (1.73)	42 (2.69)	
Outdoor & indoor air pollution exposure					
SO_2_, μg/m^3^		23.95 [16.37, 36.22]	23.77 [16.37, 34.65]	26.93 [20.29, 42.42]	<0.001*
NO_2_, μg/m^3^		30.22 [21.84, 38.12]	29.81 [21.77, 37.92]	33.83 [25.68, 42.93]	<0.001*
O_3_, μg/m^3^		81.43 [74.94, 92.30]	81.19 [74.51, 91.57]	82.53 [75.89, 93.08]	<0.001*
PM_1_, μg/m^3^		35.38 [31.53, 41.08]	35.26 [31.53, 40.92]	38.01 [32.00, 43.98]	<0.001*
PM_2.5_, μg/m^3^		52.20 [39.62, 64.34]	51.83 [39.31, 63.77]	56.69 [42.44, 76.72]	<0.001*
PM_10_, μg/m^3^		86.69 [62.68, 106.49]	85.48 [62.68, 105.37]	95.43 [75.54, 131.23]	<0.001*
Fuel	Clean fuel use	6108 (51.91)	5310 (52.03)	798 (51.12)	0.519
	Solid fuel use	5658 (48.09)	4895 (47.97)	763 (48.88)	

BMI, body mass index; SBP, systolic blood pressure; DBP, diastolic blood pressure; FBG, fast blood glucose; Tch, total cholesterol; TG, triglyceride; LDL, low density lipoprotein; HDL, high density lipoprotein; SO_2_, sulfur dioxide; NO_2_, nitrogen dioxide; O_3_, ozone; PM_1_, particulate matter with aerodynamic diameters of ≤1 μm; PM_2.5_, particulate matter with aerodynamic diameters of ≤ 2.5 μm; PM_10_, particulate matter with aerodynamic diameters of ≤ 10 μm.

^a^Data are presented as median and the interquartile range (IQR) for continuous variables and counts (percentages) for categorical variables. Examining differences between obesity and non-obesity based on Kruskal-Wallis tests for continuous variables and Chi-squared tests for categorical variables.

*P values <0.05 (two-tailed) were considered statistically significant.

The association between AP exposure and VAI quartiles is summarized in **Supplementary Table 3**. The quartile ranges were ≤ 0.96, 0.96-1.50, 1.50-2.48, and ≥ 2.48. According to the trending analysis, with the increase in VAI quartiles, BMI, systolic blood pressure, diastolic blood pressure, fasting blood glucose, TGs, and the prevalence of hypertension and diabetes increased. Moreover, we also observed a significant increase in the average AP exposure with a step increase by VAI quartiles (all P for trend< 0.001).

### 3.2 Association between outdoor AP exposure and obesity

**Figure 3:**
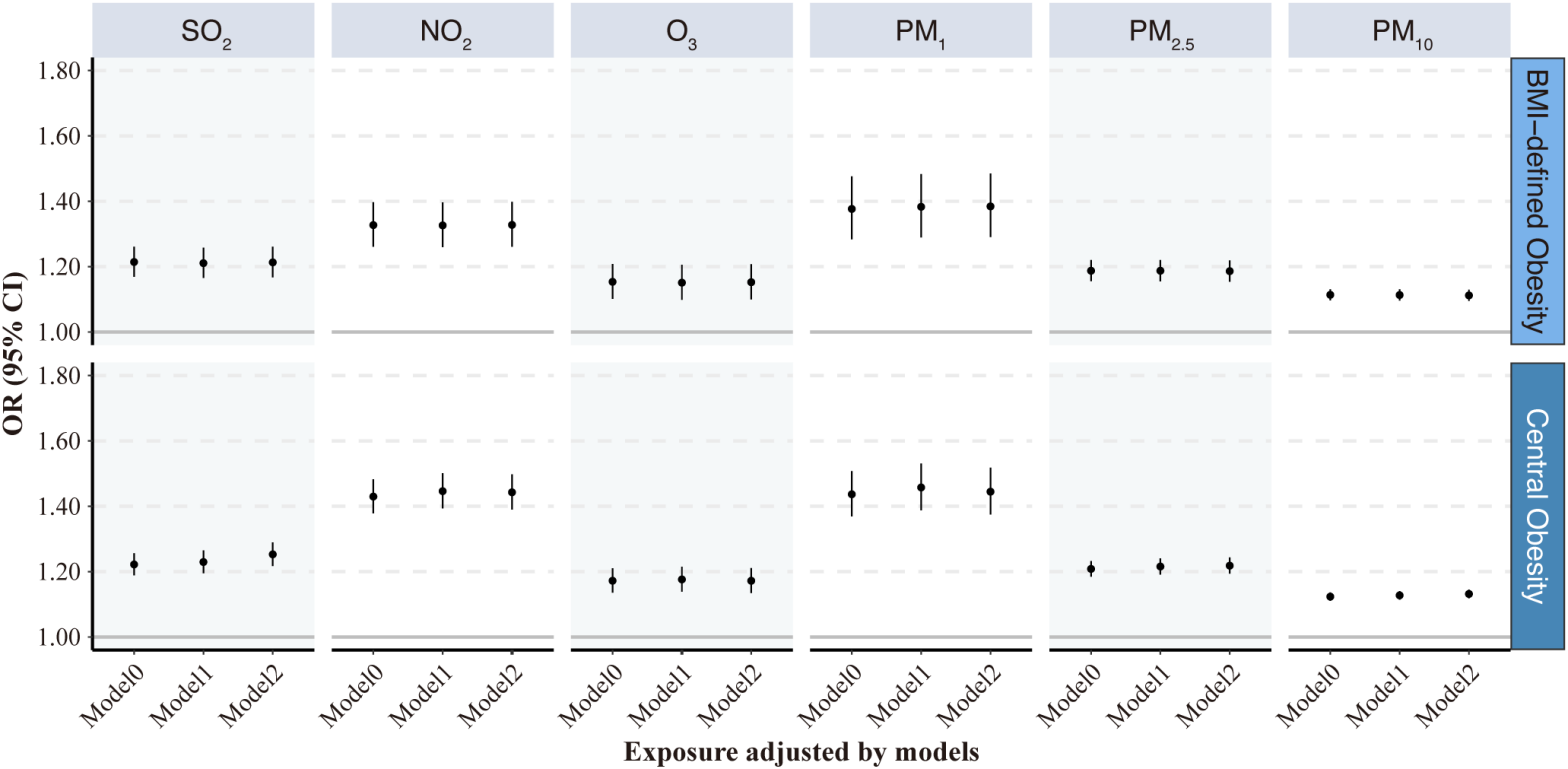
Odds ratios (95% CI) of obesity associated with each 10 μg/m^3^ increase in the 6 air pollutants. Model 0: initial crude model; Model 1: adjusted for age at baseline visit and gender; Model 2: additionally adjusted for education attainment, alcohol consumption and smoking status, and indoor fuel use. VAI, visceral adiposity index; SO_2_, sulfur dioxide; NO_2_, nitrogen dioxide; O_3_, ozone; PM_1_, particulate matter with aerodynamic diameters of ≤1 μm; PM_2.5_, particulate matter with aerodynamic diameters of ≤ 2.5 μm; PM_10_, particulate matter with aerodynamic diameters of ≤ 10 μm.

**Figure 3** presents the relationship between the prevalence of obesity and exposure to AP. The results indicate that obesity and AP exposure had positive associations in all three models (crude, Model 1, and Model 2) with varying effect sizes. In Model 2 of obesity defined by BMI, after fully adjusting for age level, sex, educational attainment, alcohol consumption, smoking status, and indoor fuel use, the ORs and 95% CI of obesity were 1.21 (1.17, 1.26), 1.33 (1.26, 1.40), 1.15 (1.10, 1.21), 1.38 (1.29, 1.48), 1.19 (1.15, 1.22), and 1.11 (1.09, 1.13) per 10 μg/m^3^ increases in SO_2_, NO_2_, O_3_, PM_1_, PM_2.5_, and PM_10_, respectively, suggesting that all 6 ambient AP exposures were significantly associated with the increased prevalence of obesity.

The results of Model 2 in obesity defined by BMI were similar to those defined by WC. When central obesity defined by WC was used as the outcome variable, the relationship between AP and obesity did not change significantly; however, AP exposure seemed to result in slightly higher ORs for central obesity defined by WC. The numerical results corresponding to the associations between 6 air pollutants and obesity presented in **Figure 3** can be found in **Supplementary Table 4**.

**Table 2** demonstrates the results of multivariate logistic regression modeling analysis between AP exposure and VAI quartiles. In Model 1, when comparing the highest quartile of VAI to the lowest quartile, the ORs for AP exposure were 1.13 (1.09, 1.18), 1.33 (1.26, 1.40), 1.13 (1.08, 1.18), 1.18 (1.10, 1.26), 1.10 (1.07, 1.13) and 1.07 (1.05, 1.09) per 10 μg/m^3^ increase in SO_2_, NO_2_, O_3_, PM_1_, PM_2.5_, and PM_10_, respectively. After additional adjustments for lifestyle risk factors, education level, and indoor fuel use, the ORs of AP exposure slightly decreased but did not change the association.

**Table 2:** Odds ratios (95% CI) of VAI associated with each 10 μg/m^3^ increase in the 6 air pollutants.

Model	Pollute	Quartile 1	Quartile 2	Quartile 3	Quartile 4
Model0	SO_2_	ref.	1.06 (1.01, 1.10)	1.14 (1.10, 1.19)	1.13 (1.09, 1.18)
	NO_2_	ref.	1.22 (1.16, 1.29)	1.33 (1.27, 1.40)	1.32 (1.25, 1.39)
	O_3_	ref.	1.06 (1.01, 1.11)	1.08 (1.04, 1.13)	1.13 (1.08, 1.18)
	PM_1_	ref.	1.11 (1.04, 1.19)	1.21 (1.13, 1.29)	1.17 (1.09, 1.24)
	PM_2.5_	ref.	1.06 (1.03, 1.09)	1.11 (1.08, 1.14)	1.10 (1.07, 1.13)
	PM_10_	ref.	1.04 (1.03, 1.06)	1.07 (1.06, 1.09)	1.07 (1.05, 1.09)
Model1	SO_2_	ref.	1.06 (1.02, 1.10)	1.14 (1.10, 1.19)	1.13 (1.09, 1.18)
	NO_2_	ref.	1.23 (1.17, 1.30)	1.34 (1.27, 1.41)	1.33 (1.26, 1.40)
	O_3_	ref.	1.06 (1.01, 1.11)	1.08 (1.04, 1.13)	1.13 (1.08, 1.18)
	PM_1_	ref.	1.12 (1.04, 1.19)	1.21 (1.13, 1.30)	1.18 (1.10, 1.26)
	PM_2.5_	ref.	1.06 (1.03, 1.09)	1.11 (1.08, 1.14)	1.10 (1.07, 1.13)
	PM_10_	ref.	1.04 (1.03, 1.06)	1.08 (1.06, 1.09)	1.07 (1.05, 1.09)
Model2	SO_2_	ref.	1.07 (1.02, 1.11)	1.16 (1.11, 1.21)	1.15 (1.10, 1.20)
	NO_2_	ref.	1.23 (1.17, 1.29)	1.34 (1.27, 1.41)	1.33 (1.26, 1.40)
	O_3_	ref.	1.06 (1.01, 1.11)	1.08 (1.03, 1.13)	1.12 (1.07, 1.17)
	PM_1_	ref.	1.10 (1.03, 1.18)	1.20 (1.12, 1.29)	1.16 (1.08, 1.24)
	PM_2.5_	ref.	1.06 (1.03, 1.09)	1.11 (1.08, 1.14)	1.10 (1.07, 1.14)
	PM_10_	ref.	1.05 (1.03, 1.06)	1.08 (1.06, 1.10)	1.07 (1.06, 1.09)

VAI, visceral adiposity index; SO_2_, sulfur dioxide; NO_2_, nitrogen dioxide; O_3_, ozone; PM_1_, particulate matter with aerodynamic diameters of ≤1 μm; PM_2.5_, particulate matter with aerodynamic diameters of ≤ 2.5 μm; PM_10_, particulate matter with aerodynamic diameters of ≤ 10 μm.

*Multiple logistic regression.

Model 0: initial crude model; Model 1: adjusted for age at baseline visit and gender; Model 2: additionally adjusted for education attainment, alcohol consumption and smoking status, and indoor fuel use.

### 3.3 Stratified analyses

The results of several stratified analyses conducted to assess the effects of AP exposure in participants with different characteristics are presented in **Table 3**, as classified by BMI. Overall, the positive association between AP exposure and the prevalence of BMI-defined obesity was not substantially modified by sex, educational attainment, smoking status, or alcohol consumption. Notably, the observed positive association of NO_2,_ PM_1_, PM_2.5_, and PM_10_ exposures was more pronounced among elderly participants (ORs: 1.50 (1.35, 1.67), 1.60 (1.37, 1.87), 1.26 (1.19, 1.33) and 1.15 (1.12, 1.19) per 10 μg/m^3^ increases in air pollutant above), while the relationship remained significant between O_3_ and both female and second-hand smokers (ORs: 1.21 (1.14, 1.28) for female and 1.28 (1.19, 1.38) for the second-hand smoker), demonstrating a strong interaction between outdoor O_3_ exposure and sex and smoking status on BMI-defined obesity. In addition, we observed that participants with lower education levels and those who were second-hand smokers or who drink more than once a month were more likely to develop BMI-defined obesity when exposed to ambient AP, despite there being no statistically significant interactions for education attainment and lifestyle risk factors.

**Table 3:** Odds ratios (95% CI) of BMI-defined obesity associated with each 10 μg/m^3^ increase of SO_2_, NO_2_, O_3_, PM_1_, PM_2.5_, PM_10_, stratified by demographic and lifestyle factors.

Effect modifiers^a^	SO_2_		NO_2_		O_3_		PM_1_		PM_2.5_		PM_10_	
	OR (95% CI)	p value^b^	OR (95% CI)	p value	OR (95% CI)	p value	OR (95% CI)	p value	OR (95% CI)	p value	OR (95% CI)	p value
**Main analyses**	1.21 (1.17, 1.26)	–	1.33 (1.26, 1.40)	–	1.15 (1.10, 1.21)	–	1.38 (1.29, 1.48)	–	1.19 (1.15, 1.22)	–	1.11 (1.10, 1.13)	–
Gender												
Male	1.21 (1.14, 1.29)	Ref.	1.27 (1.17, 1.38)	Ref.	1.07 (0.99, 1.16)	Ref.	1.39 (1.24, 1.56)	Ref.	1.18 (1.13, 1.23)	Ref.	1.11 (1.08, 1.13)	Ref.
Female	1.21 (1.15, 1.27)	0.683	1.36 (1.27, 1.45)	0.267	1.21 (1.14, 1.28)	0.026	1.38 (1.27, 1.51)	0.877	1.19 (1.15, 1.23)	0.707	1.11 (1.09, 1.14)	0.494
Age												
Middle-aged (45 - 64 years)	1.21 (1.16, 1.27)	Ref.	1.28 (1.21, 1.36)	Ref.	1.14 (1.08, 1.21)	Ref.	1.33 (1.23, 1.44)	Ref.	1.17 (1.13, 1.20)	Ref.	1.10 (1.08, 1.12)	Ref.
Elderly (65 years and above)	1.21 (1.11, 1.31)	0.745	1.50 (1.35, 1.67)	0.008	1.19 (1.08, 1.31)	0.403	1.60 (1.37, 1.87)	0.028	1.26 (1.19, 1.33)	0.032	1.15 (1.12, 1.19)	0.019
Educational attainment												
Middle school or below	1.20 (1.15, 1.25)	Ref.	1.35 (1.28, 1.43)	Ref.	1.17 (1.11, 1.23)	Ref.	1.41 (1.31, 1.52)	Ref.	1.19 (1.16, 1.23)	Ref.	1.12 (1.10, 1.14)	Ref.
High school or above	1.29 (1.15, 1.44)	0.192	1.20 (1.04, 1.37)	0.094	1.04 (0.92, 1.18)	0.104	1.24 (1.02, 1.51)	0.214	1.15 (1.06, 1.24)	0.415	1.08 (1.04, 1.13)	0.24
Smoking status												
Non-smoker	1.20 (1.12, 1.30)	Ref.	1.26 (1.14, 1.38)	Ref.	1.07 (0.99, 1.17)	Ref.	1.34 (1.17, 1.53)	Ref.	1.14 (1.09, 1.20)	Ref.	1.09 (1.06, 1.12)	Ref.
Smoker	1.19 (1.12, 1.27)	0.851	1.30 (1.19, 1.43)	0.621	1.08 (1.00, 1.17)	0.919	1.39 (1.23, 1.58)	0.739	1.20 (1.14, 1.26)	0.228	1.12 (1.09, 1.15)	0.193
Second-hand Smoker	1.23 (1.16, 1.31)	0.455	1.40 (1.29, 1.52)	0.107	1.28 (1.19, 1.38)	0.004	1.42 (1.27, 1.58)	0.593	1.21 (1.15, 1.26)	0.103	1.12 (1.10, 1.15)	0.061
Drinking status												
Non-drinker	1.21 (1.16, 1.27)	Ref.	1.34 (1.26, 1.43)	Ref.	1.19 (1.12, 1.26)	Ref.	1.40 (1.29, 1.52)	Ref.	1.20 (1.16, 1.24)	Ref.	1.12 (1.10, 1.14)	Ref.
Drink but less than once a month	1.13 (0.98, 1.30)	0.344	1.27 (1.05, 1.54)	0.58	1.11 (0.93, 1.33)	0.503	1.18 (0.92, 1.51)	0.167	1.12 (1.02, 1.24)	0.238	1.07 (1.01, 1.13)	0.159
Drink more than once a month	1.23 (1.13, 1.35)	0.801	1.29 (1.15, 1.44)	0.71	1.06 (0.96, 1.17)	0.081	1.42 (1.21, 1.66)	0.685	1.17 (1.10, 1.24)	0.544	1.11 (1.07, 1.15)	0.707

BMI, body mass index; SO_2_, sulfur dioxide; NO_2_, nitrogen dioxide; O_3_, ozone; PM_1_, particulate matter with aerodynamic diameters of ≤1 μm; PM_2.5_, particulate matter with aerodynamic diameters of ≤ 2.5 μm; PM_10_, particulate matter with aerodynamic diameters of ≤ 10 μm.

^a^The effects were estimated by logistic regression models with adjustment for gender, age, educational attainment, smoking status and drinking status. All stratified estimates were adjusted for the remaining covariates.

^b^P values represent the interaction effects between air pollutants and possible modifiers.

Similar effects were also observed in WC-defined central obesity (**Supplementary Table 5**). However, there were no significant differences in the interactions between AP exposure and central obesity among participants stratified by different characteristics.

### 3.4 Sensitivity analyses

**Table 4** displays the estimated ORs of obesity for all models. Sensitivity analysis 1 indicated that the estimated OR of obesity with AP exposure remained similar when only participants who had resided at their present residential location for over 3 years were included. Sensitivity analysis 2 found that when further including temperature and specific humidity, the ORs for obesity due to PM_1_ exposure slightly increased. Specifically, the OR was 1.59 (1.42, 1.77) vs. 1.48 (1.36, 1.61) for BMI-defined obesity and 1.57 (1.46, 1.69) vs. 1.53 (1.44, 1.62) for WC-defined central obesity. In sensitivity analysis 3, there was no significant difference in all models after adjusting for either hypertension or diabetes history. In sensitivity analysis 4, the results were consistent with the primary model when a sample with a personal history of cardiac events or stroke was restricted. Overall, sensitivity analysis suggested that the main results were robust concerning the participants.

**Table 4:** Sensitivity analyses of ORs (95% CIs) of BMI-defined obesity associated with each 10 μg/m^3^ increase in the 6 air pollutants.

	SO_2_	NO_2_	O_3_	PM_1_	PM_2.5_	PM_10_
BMI-defined obesity						
**Main analyses^a^**	1.21 (1.17, 1.26)	1.33 (1.26, 1.40)	1.15 (1.10, 1.21)	1.38 (1.29, 1.48)	1.19 (1.15, 1.22)	1.11 (1.10, 1.13)
**Additional adjustment for**						
Personal histories of hypertension and diabetes	1.20 (1.16, 1.25)	1.30 (1.23, 1.37)	1.14 (1.09, 1.20)	1.36 (1.27, 1.46)	1.17 (1.14, 1.21)	1.11 (1.09, 1.12)
Meteorological factors including temperature and relative humidity	1.12 (1.07, 1.18)	1.29 (1.21, 1.37)	1.07 (1.02, 1.13)	1.58 (1.44, 1.74)	1.19 (1.15, 1.23)	1.12 (1.10, 1.15)
**Restricted to**						
Those at the current residence >3 years	1.22 (1.17, 1.27)	1.33 (1.26, 1.40)	1.15 (1.10, 1.21)	1.40 (1.31, 1.51)	1.19 (1.16, 1.22)	1.11 (1.10, 1.13)
Those without personal histories of cardiac events or stroke	1.23 (1.18, 1.29)	1.34 (1.27, 1.42)	1.16 (1.10, 1.23)	1.47 (1.35, 1.59)	1.19 (1.16, 1.23)	1.12 (1.10, 1.14)
**Completely adjusted**	1.13 (1.06, 1.19)	1.25 (1.16, 1.34)	1.08 (1.02, 1.14)	1.54 (1.38, 1.72)	1.17 (1.12, 1.22)	1.11 (1.08, 1.14)
Central obesity						
**Main analyses^a^**	1.25 (1.22, 1.29)	1.44 (1.39, 1.50)	1.17 (1.13, 1.21)	1.44 (1.37, 1.52)	1.22 (1.19, 1.24)	1.13 (1.12, 1.15)
**Additional adjustment for**						
Personal histories of hypertension and diabetes	1.25 (1.21, 1.29)	1.42 (1.37, 1.48)	1.17 (1.13, 1.21)	1.43 (1.36, 1.51)	1.21 (1.19, 1.24)	1.13 (1.11, 1.14)
Meteorological factors including temperature and relative humidity	1.14 (1.10, 1.19)	1.41 (1.35, 1.48)	1.08 (1.05, 1.12)	1.62 (1.51, 1.73)	1.21 (1.18, 1.24)	1.14 (1.12, 1.16)
**Restricted to**						
Those at the current residence >3 years	1.26 (1.22, 1.29)	1.44 (1.39, 1.50)	1.18 (1.14, 1.22)	1.46 (1.39, 1.53)	1.22 (1.19, 1.24)	1.13 (1.12, 1.15)
Those without personal histories of cardiac events or stroke	1.27 (1.23, 1.31)	1.45 (1.39, 1.51)	1.18 (1.14, 1.22)	1.51 (1.43, 1.60)	1.22 (1.20, 1.25)	1.13 (1.12, 1.15)
**Completely adjusted**	1.16 (1.11, 1.21)	1.37 (1.30, 1.44)	1.09 (1.05, 1.13)	1.53 (1.42, 1.65)	1.19 (1.15, 1.22)	1.12 (1.10, 1.15)

^**a**^Main analyses: adjusted for gender, age, educational attainment, smoking status, drinking status and indoor fuel use.

SO_2_, sulfur dioxide; NO_2_, nitrogen dioxide; O_3_, ozone; PM_1_, particulate matter with aerodynamic diameters of ≤1 μm; PM_2.5_, particulate matter with aerodynamic diameters of ≤ 2.5 μm; PM_10_, particulate matter with aerodynamic diameters of ≤ 10 μm.

**Supplementary Figure 1** illustrates the association between obesity and AP exposure on a continuous scale using the RCS model. The spline curves indicate a significant nonlinear relationship between obesity and SO_2_, PM_2.5_, and PM_10_ exposures according to the RCS models. However, the nonlinear results of NO_2_ and PM_1_ differ between BMI-defined obesity and central obesity. Regarding the O_3_ curves, linearity of the exposure-response association was observed in both BMI-defined obesity and central obesity. As depicted in **Supplementary Figure 1A**, the risk of obesity increased with the level of SO_2_ when the SO_2_ concentration was less than 42.9 μg/m^3^ in BMI-defined obesity and 46.2 μg/m^3^ in central obesity but decreased when the SO_2_ concentration exceeded these values. With regard to the other 5 air pollutants, irrespective of the significance of the linearity effect within the observable range of the corresponding air pollution concentration, the ORs of obesity increased with the AP concentration. The results of the significance tests corresponding to **Supplementary Figure 1** are shown in **Supplementary Table 6**.

## 4 Discussion

To the best of our knowledge, this was the first study to investigate the association between different parameters of obesity and AP exposure among middle-aged and elderly individuals within the Chinese population. Our cross-sectional study of 13,077 participants found that exposure to outdoor AP, including gaseous pollutants (SO_2_, NO_2_, O_3_) and particulate matter (PM_1_, PM_2.5_, and PM_10_), was significantly linked to higher obesity risk. Moreover, we also found significant multiplicative interaction effects between O_3_ exposure and several demographic and lifestyle factors on BMI-defined obesity, including sex, age, and smoking status. Sensitivity analyses validated the findings.

We are aware that there are several relevant epidemiological studies that analyze the impact of exposure to AP on obesity risk within the Chinese population. One study enrolled 24,845 participants and suggested that exposure to high concentrations of AP is positively correlated with overweight and obesity among adults (28). However, the survey area of the respondents was limited to Liaoning, an industry-oriented province in northeast China (29), which may not reflect the nationwide relationship between AP exposure and obesity. Another study found similar results, but limited to Anhui province in eastern China and used only BMI to classify normal weight and obesity. This limitation may restrict the potential biological implications related to adipose tissues (30). Although BMI is a general measure of obesity or adiposity in the Chinese population, it does not offer details about body-fat distribution, specifically subcutaneous adiposity or visceral adiposity. Studies have shown that visceral adiposity, or the amount of fat stored around the abdominal organs, is a better indicator of cardiovascular disease than BMI alone (31, 32). Additionally, a previous study reported that Chinese populations present higher levels of visceral adiposity than white people when total body fat mass is equal (33). This could potentially explain the increased risk of cardiometabolic disease in the Chinese population with a low BMI. In addition, studies have suggested that in the absence of clinical imaging evidence, WC is currently recommended as an alternative marker for measuring ectopic fat and central obesity (34, 35). In the current study, our results showed that AP was significantly linked to a higher risk of central obesity and a higher quantile of VAI in middle-aged and elderly adults, providing a more comprehensive view of the impact of AP exposure on abdominal and visceral adipose tissue beyond BMI alone.

Our study suggested that women may suffer greater impacts of O_3_ exposure on the risk of obesity, consistent with a prior study (36). There is growing evidence reporting the sex-specific difference between AP exposure and health, although some controversy still remains over their exact biological roles (37, 38). One time-series study proposed that females were more vulnerable when exposed to AP (39). Another study based on a Chinese rural population suggested that AP exposure in females was linked to an increased prevalence of obesity (11). Previous studies reported that women may be more susceptible than men to the adverse effect of O_3_ exposure, manifested by higher rates of hospitalization and slightly higher mortality for a 10-ppb increase in 8-hour O_3_ concentration (40–42). The underlying mechanism of sex-specific differences may be partially explained by hormones and physiological fluctuations in the female reproductive cycle (43, 44). Researchers have observed that there is no appreciable interaction between O_3_ exposure and smoking (45), which is consistent with our findings. In this study, a multiplicative interaction with O_3_ exposure was observed specifically among second-hand smokers rather than smokers, which may be partially explained by the enhanced sensitivity of the lungs to O_3_ exposure following short-term sidestream cigarette smoke (46, 47). Our results showed that AP exposure had a significant effect on BMI-defined obesity in elderly adults compared with the middle-aged population. Age may affect the association between AP and obesity, and the phenomenon may be attributed to decreased physical functioning and poorer physical reserves in the elderly population (48). Elderly individuals are known to have poor immune responsiveness, which can lead to increased susceptibility, severity of diseases, and systemic inflammation when exposed to AP (49).

Although the detailed mechanism underlying AP in obesity remains unclear, several animal experiments could partially interpret the potential associations. First, previous studies have suggested that exposure to O_3_ could stimulate the synthesis and secretion of leptin by activating lipopolysaccharides (50) and the hypothalamus pituitary adrenal axis (51) and increasing the release of fasting fatty acids (52), which may be associated with subsequent obesity (53). Second, a study revealed that exposure to ambient particulate matter induced systemic inflammation, which can lead to inflammation in various organs, including the liver and adipose tissue. This inflammation can contribute to the overgrowth of adipose tissue and dysregulation of lipid homeostasis (54). Additionally, particulate matter exposure increased oxidative stress and induced Toll-like receptor 2/4-dependent inflammatory activation in the lung and further spilled over systematically, finally leading to metabolic dysfunction and obesity (14). Recent studies have also raised the hypothesis that the gut microbiota may mediate the association between AP and obesity (55–57). Briefly, AP exposure can change the composition of gut bacteria, resulting in increased proinflammatory cytokine production and a decrease in glucose intolerance, which may be linked to obesity and diabetes.

A novel finding of the current study was that exposure to PM_1_ resulted in greater harm to obesity risks than exposure to other APs presented in this study. In other words, it appears that a larger diameter of the particles corresponds to a lower health hazard. Previous studies have explored the association of health hazards and the size of particles (58, 59); however, these studies have mostly focused on PM_2.5_ and PM_10_, with insufficient evidence regarding the health implications of smaller particles such as PM_1_ (60, 61). One possible explanation for this phenomenon is that smaller particles, especially PM_1_, could more easily enter the acinar section of the respiratory tract due to their higher surface-to-volume ratio, consequently promoting oxidative stress and inflammation and further health distress (62). Another alternative explanation is that PM_1_ may contain anthropogenic toxins, such as metals, which can lead to health damage and further genetic abnormalities as well as cancer (63).

This study is subject to several limitations. First, due to the absence of more detailed addresses, the AP concentrations matched to individuals were at county levels, which prevented the assessment of occupational risks and may deviate from the ideal theoretical value. Second, we only calculated the 1-year average outdoor AP exposure for analysis in the current study, which may result in misestimation of the effects of AP on obesity. Third, as our study is based on cross-sectional data, we cannot establish causal relationships between AP and the risk of obesity. Fourth, the unavailability of more lifestyle characteristics in the CHARLS dataset might lead to an underestimate of our results. Last, this study was conducted in an Asian population, and further validation is necessary when generalizing our findings to other populations and regions.

## 5 Conclusion

In conclusion, the present study found that there is a significant association between exposure to outdoor AP and an increased risk of obesity in middle-aged and elderly Chinese populations. Our findings contribute to evidence demonstrating the detrimental impact of outdoor AP on both subcutaneous adiposity and visceral adiposity. We also observed that women and elderly individuals may be more vulnerable to AP exposure-associated obesity. Additionally, our study demonstrated that smaller diameter of the particles corresponds to more health hazard. Further prospective studies are required to confirm the results of this study.

## Data availability statement

Datasets were obtained from the China Health and Retirement Longitudinal Study (CHARLS) 2015 (available at https://charls.pku.edu.cn/); Air pollution exposures were collected from ChinaHighAirPollutants (CHAP) dataset (available at https://weijing-rs.github.io/product.html); Meteorological factors (temperature and relative humidity) were obtained from meteorological monitoring stations in the China Meteorological Administration (http://data.cma.cn/).

## Ethics statement

The studies involving humans were approved by Institutional Review Board of Peking University (Code: IRB00001052-11015). The studies were conducted in accordance with the local legislation and institutional requirements. The participants provided their written informed consent to participate in this study.

## Author contributions

JW: Conceptualization, Methodology. J-JQ: Methodology, Project administration. WP: Software, Writing - Original Draft, Data curation. YH: Visualization. ZL: Investigation, Software. HC: Supervision, Validation. MW: Writing - Review & Editing. All authors contributed to the article and approved the submitted version.

## Supplementary material

Supplementary Figure 1 Restricted cubic spline with different knots for potential nonlinear association between 6 air pollutants and obesity on a continuous scale. The ORs and 95% confidence intervals represent the predicted levels of 6 air pollutants. Analyses were fully adjusted for age level, gender, education attainment, smoking status, alcohol consumption, history of hypertension and diabetes, indoor fuel use, temperature and specific humidity. OR, odds ratio; CI, confidence interval; BMI, body-mass index; SO_2_, sulfur dioxide; NO_2_, nitrogen dioxide; O_3_, ozone; PM_1_, particulate matter with aerodynamic diameters of ≤1 μm; PM_2.5_, particulate matter with aerodynamic diameters of ≤ 2.5 μm; PM_10_, particulate matter with aerodynamic diameters of ≤ 10 μm.
