# Supplementary material for: The association between outdoor air pollution and body mass index, central obesity, and visceral adiposity index among middle-aged and elderly adults: a nationwide study in China
Source: Front Endocrinol (Lausanne). 2023 Oct 9;14:1221325. doi: 10.3389/fendo.2023.1221325 (PMC10593432; doi:10.3389/fendo.2023.1221325)
Supplement: Supplementary Figure 1 — Restricted cubic spline with different knots for potential nonlinear association between 6 air pollutants and obesity on a continuous scale. The ORs and 95% confidence intervals represent the predicted levels of 6 air pollutants. Analyses were fully adjusted for age level, gender, education attainment, smoking status, alcohol consumption, history of hypertension and diabetes, indoor fuel use, temperature and specific humidity. OR, odds ratio; CI, confidence interval; BMI, body-mass index; SO2, sulfur dioxide; NO2, nitrogen dioxide; O3, ozone; PM1, particulate matter with aerodynamic diameters of ≤1 μm; PM2.5, particulate matter with aerodynamic diameters of ≤ 2.5 μm; PM10, particulate matter with aerodynamic diameters of ≤ 10 μm. [file DataSheet_1.docx]

**Figure S1. Restricted cubic spline with different knots for potential nonlinear association between 6 air pollutants and obesity.**


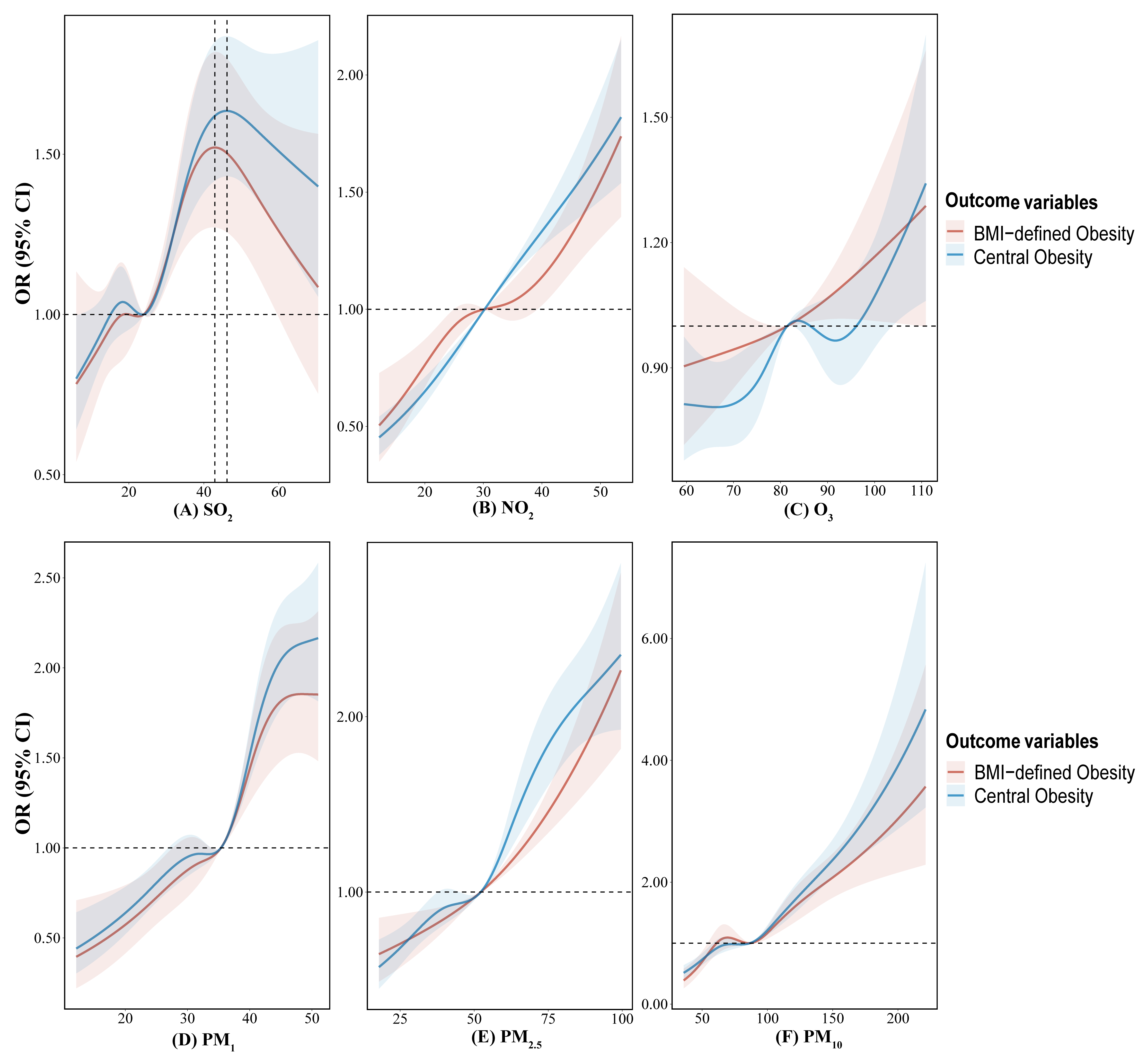


The ORs and 95% confidence intervals represent the predicted levels of 6 air pollutants. Analyses were fully adjusted for age level, gender, education attainment, smoking status, alcohol consumption, history of hypertension and diabetes, indoor fuel use, temperature and specific humidity.

**Abbreviations:** OR, odds ratio; CI, confidence interval; BMI, body-mass index; SO_2_, sulfur dioxide; NO_2_, nitrogen dioxide; O_3_, ozone; PM_1_, particulate matter with aerodynamic diameters of ≤1 μm; PM_2.5_, particulate matter with aerodynamic diameters of ≤ 2.5 μm; PM_10_, particulate matter with aerodynamic diameters of ≤ 10 μm.

**Table S1. The spatial distribution of obesity prevalence in 28 provinces of China.**

| **Province** | **BMI-defined obesity (%)** | **Central obesity (%)** |
| --- | --- | --- |
| Anhui | 13.32 | 48.65 |
| Beijing | 9.09 | 72.73 |
| Chongqing | 8.76 | 27.74 |
| Fujian | 7.07 | 34.73 |
| Gansu | 9.54 | 40.13 |
| Guangdong | 6.32 | 40.75 |
| Guangxi | 7.05 | 30.08 |
| Guizhou | 5.88 | 26.47 |
| Hebei | 21.62 | 63.91 |
| Heilongjiang | 18.06 | 58.59 |
| Henan | 18.49 | 58.31 |
| Hubei | 6.95 | 36.09 |
| Hunan | 8.32 | 34.82 |
| Jiangsu | 13.43 | 53.89 |
| Jiangxi | 8.22 | 41.45 |
| Jilin | 15.21 | 55.13 |
| Liaoning | 13.26 | 49.60 |
| Neimenggu | 21.64 | 53.92 |
| Qinghai | 12.75 | 43.14 |
| Shaanxi | 11.32 | 41.34 |
| Shandong | 21.92 | 61.04 |
| Shanghai | 8.89 | 51.11 |
| Shanxi | 14.67 | 49.87 |
| Sichuan | 11.66 | 39.66 |
| Tianjin | 26.87 | 73.13 |
| Xinjiang | 25.93 | 79.63 |
| Yunnan | 6.06 | 31.27 |
| Zhejiang | 6.90 | 38.31 |

**Abbreviation:** BMI, body mass index;

**Table S2. Characteristics of the participants according to the presence of central obesity.**

| **Characteristics** | **level** | **Overall (n = 11,766)** | **Non-obesity (n = 6,251)** | **Obesity (n = 5,515)** | **p value** |
| --- | --- | --- | --- | --- | --- |
| **Demographics** |  |  |  |  |  |
| Age, years |  | 60.00 [52.00, 67.00] | 60.00 [52.00, 67.00] | 59.00 [52.00, 66.00] | <0.001* |
| Age group | Middle-aged (45 - 64 years) | 7985 (67.87) | 4116 (65.85) | 3869 (70.15) | <0.001* |
|  | Elderly (65 years and above) | 3781 (32.13) | 2135 (34.15) | 1646 (29.85) |  |
| Gender | Male | 5553 (47.20) | 3550 (56.79) | 2003 (36.32) | <0.001* |
|  | Female | 6213 (52.80) | 2701 (43.21) | 3512 (63.68) |  |
| Education level | Middle school or below | 8989 (88.01) | 4785 (87.86) | 4204 (88.17) | 0.654 |
|  | High school or above | 1225 (11.99) | 661 (12.14) | 564 (11.83) |  |
| Residence | Rural | 7288 (61.94) | 4211 (67.37) | 3077 (55.79) | <0.001* |
|  | Urban | 4478 (38.06) | 2040 (32.63) | 2438 (44.21) |  |
| **Lifestyle behaviors** |  |  |  |  |  |
| Smoking Status | Non-smoker | 3132 (26.62) | 1531 (24.49) | 1601 (29.03) | <0.001* |
|  | Smoker | 4877 (41.45) | 3088 (49.40) | 1789 (32.44) |  |
|  | Second-hand Smoker | 3757 (31.93) | 1632 (26.11) | 2125 (38.53) |  |
| Drinking Status | Non-drinker | 7617 (64.74) | 3791 (60.65) | 3826 (69.37) | <0.001* |
|  | Drink but less than once a month | 1033 (8.78) | 576 (9.21) | 457 (8.29) |  |
|  | Drink more than once a month | 3116 (26.48) | 1884 (30.14) | 1232 (22.34) |  |
| **Clinical characteristics** |  |  |  |  |  |
| BMI group | < 24 kg/m^2^ | 6288 (53.44) | 5328 (85.23) | 960 (17.41) | <0.001* |
|  | ≥ 24 kg/m^2^ | 5478 (46.56) | 923 (14.77) | 4555 (82.59) |  |
| SBP, mmHg |  | 126.67 [114.33, 141.33] | 123.33 [112.00, 137.67] | 130.00 [118.33, 144.33] | <0.001* |
| DBP, mmHg |  | 74.67 [67.67, 82.67] | 73.00 [66.00, 80.50] | 77.00 [70.00, 84.67] | <0.001* |
| Pulse, bpm |  | 73.00 [66.33, 80.00] | 72.67 [66.00, 79.67] | 73.67 [67.00, 80.33] | <0.001* |
| BMI, kg/m^2^ |  | 23.67 [21.35, 26.25] | 21.61 [19.93, 23.20] | 26.28 [24.61, 28.28] | <0.001* |
| Waistline, cm |  | 86.05 [79.00, 93.40] | 79.40 [74.10, 83.40] | 94.00 [90.20, 99.00] | <0.001* |
| **Laboratory measures** |  |  |  |  |  |
| FBG, mmol/L |  | 5.31 [4.90, 5.91] | 5.21 [4.80, 5.71] | 5.51 [5.01, 6.21] | <0.001* |
| Tch, mmol/L |  | 4.68 [4.12, 5.32] | 4.58 [4.03, 5.22] | 4.81 [4.25, 5.45] | <0.001* |
| TG, mmol/L |  | 1.30 [0.94, 1.93] | 1.09 [0.83, 1.56] | 1.61 [1.14, 2.33] | <0.001* |
| LDL, mmol/L |  | 2.61 [2.14, 3.10] | 2.53 [2.08, 3.01] | 2.69 [2.21, 3.19] | <0.001* |
| HDL, mmol/L |  | 1.29 [1.12, 1.49] | 1.36 [1.17, 1.57] | 1.23 [1.07, 1.40] | <0.001* |
| **Comorbidities** |  |  |  |  |  |
| Hypertension | No | 8508 (72.31) | 5036 (80.56) | 3472 (62.96) | <0.001* |
|  | Yes | 3258 (27.69) | 1215 (19.44) | 2043 (37.04) |  |
| Diabetes | No | 10895 (92.60) | 5978 (95.63) | 4917 (89.16) | <0.001* |
|  | Yes | 871 (7.40) | 273 (4.37) | 598 (10.84) |  |
| Cardiac disease | No | 10193 (86.63) | 5636 (90.16) | 4557 (82.63) | <0.001* |
|  | Yes | 1573 (13.37) | 615 (9.84) | 958 (17.37) |  |
| Stroke | No | 11547 (98.14) | 6150 (98.38) | 5397 (97.86) | 0.042* |
|  | Yes | 219 (1.86) | 101 (1.62) | 118 (2.14) |  |
| **Outdoor & indoor air pollution exposure** |  |  |  |  |  |
| SO_2_, μg/m^3^ |  | 23.95 [16.37, 36.22] | 22.85 [15.96, 31.27] | 25.97 [19.40, 40.17] | <0.001* |
| NO_2_, μg/m^3^ |  | 30.22 [21.84, 38.12] | 27.90 [20.83, 35.97] | 33.57 [24.77, 42.63] | <0.001* |
| O_3_, μg/m^3^ |  | 81.43 [74.94, 92.30] | 79.90 [73.67, 90.49] | 82.52 [75.61, 92.74] | <0.001* |
| PM_1_, μg/m^3^ |  | 35.38 [31.53, 41.08] | 34.11 [31.37, 39.25] | 36.32 [31.93, 42.25] | <0.001* |
| PM_2.5_, μg/m^3^ |  | 52.20 [39.62, 64.34] | 50.06 [38.34, 58.12] | 55.86 [41.63, 74.06] | <0.001* |
| PM_10_, μg/m^3^ |  | 86.69 [62.68, 106.49] | 83.33 [61.10, 100.13] | 92.03 [73.29, 127.73] | <0.001* |
| Fuel | Clean fuel use | 6108 (51.91) | 3090 (49.43) | 3018 (54.72) | <0.001* |
|  | Solid fuel use | 5658 (48.09) | 3161 (50.57) | 2497 (45.28) |  |

**Abbreviations:** BMI, body mass index; SBP, systolic blood pressure; DBP, diastolic blood pressure; FBG, fast blood glucose; Tch, total cholesterol; TG, triglyceride; LDL, low density lipoprotein; HDL, high density lipoprotein; SO_2_, sulfur dioxide; NO_2_, nitrogen dioxide; O_3_, ozone; PM_1_, particulate matter with aerodynamic diameters of ≤1 μm; PM_2.5_, particulate matter with aerodynamic diameters of ≤ 2.5 μm; PM_10_, particulate matter with aerodynamic diameters of ≤ 10 μm;

^a^Data are presented as median and the interquartile range (IQR) for continuous variables and counts (percentages) for categorical variables. Examining differences between obesity and non-obesity based on Kruskal-Wallis tests for continuous variables and Chi-squared tests for categorical variables.

^*^P values <0.05 (two-tailed) were considered statistically significant.

**Table S3.** **Characteristics of the participants according to the quantile of VAI.**

| **Characteristics** | **level** | **Overall  (n = 11,766)** | **Quartile 1**  **(0,0.96]  (n = 2,905)** | **Quartile 2 (0.96,1.50]  (n = 2,929)** | **Quartile 3 (1.50,2.48]  (n = 2,942)** | **Quartile 4 (2.48,39.8]  (n = 2,990)** | **p value** |
| --- | --- | --- | --- | --- | --- | --- | --- |
| **Demographics** |  |  |  |  |  |  |  |
| Age, years |  | 60.00 [52.00, 67.00] | 61.00 [53.00, 68.00] | 60.00 [52.00, 67.00] | 59.00 [52.00, 66.00] | 59.00 [52.00, 65.75] | <0.001* |
| Age group | Middle-aged (45 - 64 years) | 7985 (67.87) | 1838 (63.27) | 1967 (67.16) | 2036 (69.20) | 2144 (71.71) | <0.001* |
|  | Elderly (65 years and above) | 3781 (32.13) | 1067 (36.73) | 962 (32.84) | 906 (30.80) | 846 (28.29) |  |
| Gender | Male | 5553 (47.20) | 1910 (65.75) | 1387 (47.35) | 1187 (40.35) | 1069 (35.75) | <0.001* |
|  | Female | 6213 (52.80) | 995 (34.25) | 1542 (52.65) | 1755 (59.65) | 1921 (64.25) |  |
| Education level | Middle school or below | 8989 (88.01) | 2231 (88.81) | 2268 (88.77) | 2201 (86.45) | 2289 (88.00) | 0.032* |
|  | High school or above | 1225 (11.99) | 281 (11.19) | 287 (11.23) | 345 (13.55) | 312 (12.00) |  |
| Residence | Rural | 7288 (61.94) | 2049 (70.53) | 1865 (63.67) | 1740 (59.14) | 1634 (54.65) | <0.001* |
|  | Urban | 4478 (38.06) | 856 (29.47) | 1064 (36.33) | 1202 (40.86) | 1356 (45.35) |  |
| **Lifestyle behaviors** |  |  |  |  |  |  |  |
| Smoking Status | Non-smoker | 3132 (26.62) | 728 (25.06) | 793 (27.07) | 790 (26.85) | 821 (27.46) | <0.001* |
|  | Smoker | 4877 (41.45) | 1567 (53.94) | 1196 (40.83) | 1100 (37.39) | 1014 (33.91) |  |
|  | Second-hand Smoker | 3757 (31.93) | 610 (21.00) | 940 (32.09) | 1052 (35.76) | 1155 (38.63) |  |
| Drinking Status | Non-drinker | 7617 (64.74) | 1552 (53.43) | 1897 (64.77) | 2015 (68.49) | 2153 (72.01) | <0.001* |
|  | Drink but less than once a month | 1033 (8.78) | 254 (8.74) | 272 (9.29) | 266 (9.04) | 241 (8.06) |  |
|  | Drink more than once a month | 3116 (26.48) | 1099 (37.83) | 760 (25.95) | 661 (22.47) | 596 (19.93) |  |
| **Clinical characteristics** |  |  |  |  |  |  |  |
| BMI group | < 24 kg/m^2^ | 6288 (53.44) | 2289 (78.80) | 1743 (59.51) | 1299 (44.15) | 957 (32.01) | <0.001* |
|  | ≥ 24 kg/m^2^ | 5478 (46.56) | 616 (21.20) | 1186 (40.49) | 1643 (55.85) | 2033 (67.99) |  |
| SBP, mmHg |  | 126.67 [114.33, 141.33] | 124.33 [112.00, 139.33] | 126.00 [113.67, 140.33] | 126.67 [114.67, 142.00] | 129.33 [117.33, 143.50] | <0.001* |
| DBP, mmHg |  | 74.67 [67.67, 82.67] | 73.00 [66.00, 81.00] | 74.33 [67.33, 81.67] | 75.00 [68.33, 83.00] | 76.67 [69.33, 84.33] | <0.001* |
| Pulse, bpm |  | 73.00 [66.33, 80.00] | 72.00 [65.33, 79.08] | 72.33 [66.00, 79.00] | 73.33 [67.00, 80.33] | 74.67 [68.00, 82.00] | <0.001* |
| BMI, kg/m^2^ |  | 23.67 [21.35, 26.25] | 21.62 [19.78, 23.57] | 23.19 [21.00, 25.50] | 24.46 [22.27, 26.73] | 25.54 [23.29, 27.94] | <0.001* |
| Waistline, cm |  | 86.05 [79.00, 93.40] | 78.60 [72.50, 84.60] | 84.50 [78.30, 91.40] | 88.60 [82.60, 95.00] | 92.00 [86.00, 98.50] | <0.001* |
| **Laboratory measures** |  |  |  |  |  |  |  |
| FBG, mmol/L |  | 5.31 [4.90, 5.91] | 5.11 [4.70, 5.51] | 5.21 [4.80, 5.71] | 5.40 [4.90, 5.91] | 5.61 [5.11, 6.61] | <0.001* |
| Tch, mmol/L |  | 4.68 [4.12, 5.32] | 4.51 [4.00, 5.09] | 4.60 [4.05, 5.19] | 4.76 [4.19, 5.38] | 4.91 [4.29, 5.59] | <0.001* |
| TG, mmol/L |  | 1.30 [0.94, 1.93] | 0.78 [0.67, 0.91] | 1.08 [0.96, 1.24] | 1.54 [1.32, 1.76] | 2.59 [2.09, 3.45] | <0.001* |
| LDL, mmol/L |  | 2.61 [2.14, 3.10] | 2.47 [2.05, 2.94] | 2.68 [2.24, 3.16] | 2.76 [2.29, 3.26] | 2.49 [2.02, 3.01] | <0.001* |
| HDL, mmol/L |  | 1.29 [1.12, 1.49] | 1.53 [1.36, 1.76] | 1.35 [1.20, 1.51] | 1.25 [1.10, 1.39] | 1.11 [0.99, 1.24] | <0.001* |
| **Comorbidities** |  |  |  |  |  |  |  |
| Hypertension | No | 8508 (72.31) | 2320 (79.86) | 2209 (75.42) | 2078 (70.63) | 1901 (63.58) | <0.001* |
|  | Yes | 3258 (27.69) | 585 (20.14) | 720 (24.58) | 864 (29.37) | 1089 (36.42) |  |
| Diabetes | No | 10895 (92.60) | 2801 (96.42) | 2763 (94.33) | 2707 (92.01) | 2624 (87.76) | <0.001* |
|  | Yes | 871 (7.40) | 104 (3.58) | 166 (5.67) | 235 (7.99) | 366 (12.24) |  |
| Cardiac disease | No | 10193 (86.63) | 2631 (90.57) | 2582 (88.15) | 2503 (85.08) | 2477 (82.84) | <0.001* |
|  | Yes | 1573 (13.37) | 274 (9.43) | 347 (11.85) | 439 (14.92) | 513 (17.16) |  |
| Stroke | No | 11547 (98.14) | 2860 (98.45) | 2884 (98.46) | 2880 (97.89) | 2923 (97.76) | 0.087 |
|  | Yes | 219 (1.86) | 45 (1.55) | 45 (1.54) | 62 (2.11) | 67 (2.24) |  |
| **Outdoor & indoor air pollution exposure** |  |  |  |  |  |  |  |
| SO_2_, μg/m^3^ |  | 23.95 [16.37, 36.22] | 22.98 [16.37, 32.85] | 23.77 [16.37, 34.65] | 24.34 [16.56, 37.69] | 25.11 [17.32, 37.88] | <0.001* |
| NO_2_, μg/m^3^ |  | 30.22 [21.84, 38.12] | 27.83 [20.76, 35.85] | 30.22 [21.84, 38.57] | 31.20 [23.45, 40.41] | 31.86 [23.45, 40.47] | <0.001* |
| O_3_, μg/m^3^ |  | 81.43 [74.94, 92.30] | 80.96 [73.04, 90.49] | 81.19 [74.94, 92.30] | 81.43 [75.07, 92.44] | 82.52 [75.11, 92.48] | <0.001* |
| PM_1_, μg/m^3^ |  | 35.38 [31.53, 41.08] | 35.38 [31.39, 39.27] | 35.37 [31.58, 41.08] | 35.74 [31.75, 41.29] | 35.37 [31.39, 41.29] | <0.001* |
| PM_2.5_, μg/m^3^ |  | 52.20 [39.62, 64.34] | 49.16 [38.94, 62.74] | 52.01 [39.31, 64.34] | 53.13 [40.25, 65.80] | 53.44 [40.12, 66.43] | <0.001* |
| PM_10_, μg/m^3^ |  | 86.69 [62.68, 106.49] | 82.82 [62.28, 102.78] | 86.69 [62.68, 106.49] | 88.83 [66.91, 109.57] | 88.57 [64.39, 111.45] | <0.001* |
| Fuel | Clean fuel use | 6108 (51.91) | 1378 (47.44) | 1526 (52.10) | 1566 (53.23) | 1638 (54.78) | <0.001* |
|  | Solid fuel use | 5658 (48.09) | 1527 (52.56) | 1403 (47.90) | 1376 (46.77) | 1352 (45.22) |  |

**Abbreviations:** VAI, visceral adiposity index, BMI, body mass index; SBP, systolic blood pressure; DBP, diastolic blood pressure; FBG, fast blood glucose; Tch, total cholesterol; TG, triglyceride; LDL, low density lipoprotein; HDL, high density lipoprotein; SO_2_, sulfur dioxide; NO_2_, nitrogen dioxide; O_3_, ozone; PM_1_, particulate matter with aerodynamic diameters of ≤1 μm; PM_2.5_, particulate matter with aerodynamic diameters of ≤ 2.5 μm; PM_10_, particulate matter with aerodynamic diameters of ≤ 10 μm;

^a^Data are presented as median and the interquartile range (IQR) for continuous variables and counts (percentages) for categorical variables. Examining differences between obesity and non-obesity based on Kruskal-Wallis tests for continuous variables and Chi-squared tests for categorical variables.

^*^P values <0.05 (two-tailed) were considered statistically significant.

**Table S4.** **Odds ratios (95% CI) of obesity associated with each 10 μg/m^3^ increase of the 6 air pollutants.**

| **Model** | **Air Pollutants** | **BMI-defined Obesity** | **Central Obesity** |
| --- | --- | --- | --- |
|  |  | OR (95%CI) | |
| Model0 | SO_2_ | 1.21 (1.17, 1.26) | 1.22 (1.19, 1.26) |
|  | NO_2_ | 1.33 (1.26, 1.40) | 1.43 (1.38, 1.48) |
|  | O_3_ | 1.15 (1.10, 1.21) | 1.17 (1.14, 1.21) |
|  | PM_1_ | 1.38 (1.28, 1.48) | 1.44 (1.37, 1.51) |
|  | PM_2.5_ | 1.19 (1.16, 1.22) | 1.21 (1.18, 1.23) |
|  | PM_10_ | 1.11 (1.10, 1.13) | 1.12 (1.11, 1.14) |
| Model1 | SO_2_ | 1.21 (1.17, 1.26) | 1.23 (1.19, 1.26) |
|  | NO_2_ | 1.33 (1.26, 1.40) | 1.45 (1.39, 1.50) |
|  | O_3_ | 1.15 (1.10, 1.21) | 1.18 (1.14, 1.21) |
|  | PM_1_ | 1.38 (1.29, 1.48) | 1.46 (1.39, 1.53) |
|  | PM_2.5_ | 1.19 (1.16, 1.22) | 1.22 (1.19, 1.24) |
|  | PM_10_ | 1.11 (1.10, 1.13) | 1.13 (1.11, 1.14) |
| Model2 | SO_2_ | 1.21 (1.17, 1.26) | 1.25 (1.22, 1.29) |
|  | NO_2_ | 1.33 (1.26, 1.40) | 1.44 (1.39, 1.50) |
|  | O_3_ | 1.15 (1.10, 1.21) | 1.17 (1.13, 1.21) |
|  | PM_1_ | 1.38 (1.29, 1.48) | 1.44 (1.37, 1.52) |
|  | PM_2.5_ | 1.19 (1.15, 1.22) | 1.22 (1.19, 1.24) |
|  | PM_10_ | 1.11 (1.10, 1.13) | 1.13 (1.12, 1.15) |

**Abbreviations:** BMI, body mass index; SO_2_, sulfur dioxide; NO_2_, nitrogen dioxide; O_3_, ozone; PM_1_, particulate matter with aerodynamic diameters of ≤1 μm; PM_2.5_, particulate matter with aerodynamic diameters of ≤ 2.5 μm; PM_10_, particulate matter with aerodynamic diameters of ≤ 10 μm;

Model 0: initial crude model; Model 1: adjusted for age at baseline visit and gender; Model 2: additionally adjusted for education attainment, alcohol consumption and smoking status, and indoor fuel use.

**Table S5. Odds ratios (95% CI) of central obesity associated with each 10 μg/m3 increase of SO_2_, NO_2_, O_3_, PM_1_, PM_2.5_, PM_10_, stratified by demographic and lifestyle factors.**

| **Effect modifiers^a^** | **SO_2_** | |  | **NO_2_** | |  | **O_3_** | |  | **PM_1_** | |  | **PM_2.5_** | |  | **PM_10_** | |
| --- | --- | --- | --- | --- | --- | --- | --- | --- | --- | --- | --- | --- | --- | --- | --- | --- | --- |
|  | **OR (95% CI)** | **p value^b^** |  | **OR (95% CI)** | **p value** |  | **OR (95% CI)** | **p value** |  | **OR (95% CI)** | **p value** |  | **OR (95% CI)** | **p value** |  | **OR (95% CI)** | **p value** |
| **Main analyses** | 1.25 (1.22, 1.29) | - |  | 1.44 (1.39, 1.50) | - |  | 1.17 (1.13, 1.21) | - |  | 1.44 (1.37, 1.52) | - |  | 1.22 (1.19, 1.24) | - |  | 1.13 (1.12, 1.15) | - |
| **Gender** |  |  |  |  |  |  |  |  |  |  |  |  |  |  |  |  |  |
| Male | 1.26 (1.21, 1.31) | Ref. |  | 1.43 (1.35, 1.51) | Ref. |  | 1.17 (1.12, 1.23) | Ref. |  | 1.45 (1.35, 1.57) | Ref. |  | 1.22 (1.19, 1.26) | Ref. |  | 1.13 (1.11, 1.15) | Ref. |
| Female | 1.24 (1.19, 1.29) | 0.734 |  | 1.45 (1.38, 1.53) | 0.704 |  | 1.17 (1.12, 1.22) | 0.781 |  | 1.44 (1.35, 1.54) | 0.708 |  | 1.21 (1.18, 1.25) | 0.958 |  | 1.13 (1.11, 1.15) | 0.583 |
| **Age** |  |  |  |  |  |  |  |  |  |  |  |  |  |  |  |  |  |
| Middle-aged (45 - 64 years) | 1.24 (1.20, 1.29) | Ref. |  | 1.41 (1.35, 1.48) | Ref. |  | 1.18 (1.13, 1.23) | Ref. |  | 1.41 (1.33, 1.49) | Ref. |  | 1.20 (1.18, 1.23) | Ref. |  | 1.12 (1.11, 1.14) | Ref. |
| Elderly (65 years and above) | 1.27 (1.21, 1.34) | 0.952 |  | 1.52 (1.42, 1.63) | 0.124 |  | 1.16 (1.10, 1.23) | 0.674 |  | 1.56 (1.42, 1.72) | 0.092 |  | 1.26 (1.21, 1.31) | 0.188 |  | 1.15 (1.13, 1.18) | 0.311 |
| **Educational attainment** | |  |  |  |  |  |  |  |  |  |  |  |  |  |  |  |  |
| Middle school or below | 1.25 (1.22, 1.29) | Ref. |  | 1.46 (1.40, 1.52) | Ref. |  | 1.19 (1.15, 1.23) | Ref. |  | 1.45 (1.37, 1.53) | Ref. |  | 1.22 (1.19, 1.25) | Ref. |  | 1.13 (1.12, 1.15) | Ref. |
| High school or above | 1.24 (1.15, 1.34) | 1 |  | 1.35 (1.23, 1.49) | 0.163 |  | 1.09 (1.00, 1.19) | 0.092 |  | 1.43 (1.25, 1.63) | 0.932 |  | 1.21 (1.15, 1.28) | 0.946 |  | 1.13 (1.09, 1.16) | 0.863 |
| **Smoking status** | |  |  |  |  |  |  |  |  |  |  |  |  |  |  |  |  |
| Non-smoker | 1.29 (1.21, 1.37) | Ref. |  | 1.47 (1.37, 1.58) | Ref. |  | 1.16 (1.09, 1.24) | Ref. |  | 1.46 (1.33, 1.61) | Ref. |  | 1.23 (1.18, 1.28) | Ref. |  | 1.14 (1.11, 1.16) | Ref. |
| Smoker | 1.24 (1.19, 1.30) | 0.164 |  | 1.44 (1.36, 1.53) | 0.643 |  | 1.18 (1.12, 1.24) | 0.717 |  | 1.45 (1.34, 1.57) | 0.916 |  | 1.23 (1.19, 1.27) | 0.783 |  | 1.14 (1.11, 1.16) | 0.741 |
| Second-hand Smoker | 1.24 (1.18, 1.31) | 0.55 |  | 1.42 (1.33, 1.52) | 0.484 |  | 1.18 (1.11, 1.25) | 0.746 |  | 1.43 (1.31, 1.56) | 0.694 |  | 1.20 (1.16, 1.25) | 0.508 |  | 1.12 (1.10, 1.15) | 0.58 |
| **Drinking status** | |  |  |  |  |  |  |  |  |  |  |  |  |  |  |  |  |
| Non-drinker | 1.29 (1.21, 1.37) | Ref. |  | 1.44 (1.38, 1.51) | Ref. |  | 1.19 (1.14, 1.24) | Ref. |  | 1.45 (1.37, 1.54) | Ref. |  | 1.22 (1.19, 1.25) | Ref. |  | 1.13 (1.12, 1.15) | Ref. |
| Drink but less than once a month | 1.24 (1.19, 1.30) | 0.164 |  | 1.42 (1.25, 1.62) | 1 |  | 1.20 (1.07, 1.36) | 0.78 |  | 1.38 (1.17, 1.64) | 0.746 |  | 1.20 (1.11, 1.28) | 0.691 |  | 1.11 (1.07, 1.16) | 0.459 |
| Drink more than once a month | 1.24 (1.18, 1.31) | 0.55 |  | 1.43 (1.33, 1.54) | 0.994 |  | 1.12 (1.06, 1.19) | 0.213 |  | 1.43 (1.29, 1.58) | 0.969 |  | 1.21 (1.16, 1.26) | 0.776 |  | 1.13 (1.10, 1.16) | 0.892 |

**Abbreviations:** BMI, body mass index; SO_2_, sulfur dioxide; NO_2_, nitrogen dioxide; O_3_, ozone; PM_1_, particulate matter with aerodynamic diameters of ≤1 μm; PM_2.5_, particulate matter with aerodynamic diameters of ≤ 2.5 μm; PM_10_, particulate matter with aerodynamic diameters of ≤ 10 μm;

^a^The effects were estimated by logistic regression models with adjustment for gender, age, educational attainment, smoking status and drinking status. All stratified estimates were adjusted for the remaining covariates.

^b^P values represent the interaction effects between air pollutants and possible modifiers.

**Table S6. Detailed results of restricted cubic spline with different knots for potential nonlinear association between 6 air pollutants and obesity.**

| **Air Pollutants^a^** | **Pollutants** | **P value_curve_** | **P value_nonlinear_** | **knots** |
| --- | --- | --- | --- | --- |
| BMI-defined Obesity | SO_2_ | 0 | 0.006* | 5 |
|  | NO_2_ | 0 | 0.152 | 4 |
|  | O_3_ | 0.022 | 0.634 | 3 |
|  | PM_1_ | 0 | 0.078 | 5 |
|  | PM_2.5_ | 0 | 0.386 | 3 |
|  | PM_10_ | 0 | 0.007* | 5 |
| Central Obesity | SO_2_ | 0 | 0.001* | 5 |
|  | NO_2_ | 0 | 0.011* | 3 |
|  | O_3_ | 0 | 0.187 | 5 |
|  | PM_1_ | 0 | < 0.001* | 5 |
|  | PM_2.5_ | 0 | 0.021* | 5 |
|  | PM_10_ | 0 | 0.007* | 5 |

**Abbreviations:** BMI, body mass index; SO_2_, sulfur dioxide; NO_2_, nitrogen dioxide; O_3_, ozone; PM_1_, particulate matter with aerodynamic diameters of ≤1 μm; PM_2.5_, particulate matter with aerodynamic diameters of ≤ 2.5 μm; PM_10_, particulate matter with aerodynamic diameters of ≤ 10 μm;

^a^The effects were estimated by logistic regression-based restricted cubic spline model with adjustment for age level, gender, education attainment, smoking status, alcohol consumption, history of hypertension and diabetes, indoor fuel use, temperature and specific humidity.
