# Supplementary material for: The association between outdoor air pollution and body mass index, central obesity, and visceral adiposity index among middle-aged and elderly adults: a nationwide study in China
Source: Front Endocrinol (Lausanne). 2023 Oct 9;14:1221325. doi: 10.3389/fendo.2023.1221325 (PMC10593432; doi:10.3389/fendo.2023.1221325)
Supplement: Supplementary file 2 [file DataSheet_2.docx]

**The association between outdoor air pollution and body mass index, central obesity, and visceral adiposity index among middle-aged and elderly adults: A nationwide study in China**

Table of contents

Supplementary methods…………………………………………………………2

**Supplementary Methods**

***Sampling method***

The China Health and Retirement Longitudinal Study (CHARLS) is a longitudinal study of individuals over age 45 in China. It was designed to better understand the socioeconomic determinants and consequences of aging and there are already numerous published articles [1-3]. According to the codebook of CHARLS, the sampling method of CHARLS was selected as part of a stratified, multistage probability design. Specifically, the first component of this sampling framework was the probability proportion to size (PPS) sampling of all county-level unit except for Tibet after stratifying by region, characteristic of the county (urban or rural), and per-capita gross domestic product (GDP). Households were selected within PSUs using a CHARLS-designed mapping/listing software (CHARLS-GIS) that uses Google Earth images to list all dwelling units in all residential buildings to create sampling frames. If the sampled household had occupants older than 40, one of them was randomly selected. If the selected person was aged 45 or older, they became a respondent. If the selected person was between age 40 and 44, they were reserved as a refreshment sample.

More detailed method is available at https://g2aging.org/downloads.

***Variables***

The questionnaires and physical examinations administered to CHARLS participants have been described previously [4]. Education attainments were initially defined according to the question “***BD001_W2_4*** *Have your highest level of education changed from last wave? If so, what’s the highest level of education you have attained now? (not including adult education)*”, and the answers using the following codes:

1.No Formal Education (Illiterate), 2. Did Not Finish Primary School but can Read, 3. Sishu (Private Tutoring), 4.Elementary School, 5.Middle School, 6.High School, 7.Vocational School, 8.Two/three-year college, 9.College Grad and 10.Post-graduate degree. During the actual analysis, the educational level was grouped as “‘middle school or below’ and ‘high school or above’”, which is a simplified version of the initial classification. Middle school refers to the educational stage typically spanning grades 7 to 9, corresponding to approximately three years of schooling. High school, on the other hand, generally includes grades 10 to 12, representing an additional three years of education following middle school.

1. Gong, J., et al., *Nowcasting and forecasting the care needs of the older population in China: analysis of data from the China Health and Retirement Longitudinal Study (CHARLS).* Lancet Public Health, 2022. **7**(12): p. e1005-e1013.

2. Gao, Y., et al., *The Effect of Activity Participation in Middle-Aged and Older People on the Trajectory of Depression in Later Life: National Cohort Study.* JMIR Public Health Surveill, 2023. **9**: p. e44682.

3. Lu, X., Y. Yao, and Y. Jin, *Digital exclusion and functional dependence in older people: Findings from five longitudinal cohort studies.* EClinicalMedicine, 2022. **54**: p. 101708.

4. Zhao, Y., et al., *Cohort profile: the China health and retirement longitudinal study (CHARLS).* International journal of epidemiology, 2014. **43**(1): p. 61-68.
